# Development and validation of a risk prediction model for multidrug-resistant organisms infection in diabetic foot ulcer patients

**DOI:** 10.3389/fendo.2025.1609128

**Published:** 2025-12-11

**Authors:** Jinghang Zhang, Xuemei Li, Bai Chang, Yongmei Li, Baocheng Chang

**Affiliations:** NHC Key Lab of Hormones and Development and Tianjin Key Lab of Metabolic Diseases, Tianjin Medical University Chu Hsien-I Memorial Hospital & Institute of Endocrinology, Tianjin, China

**Keywords:** diabetic foot ulcer, multidrug-resistant organisms, infection, risk factors, nomogram

## Abstract

**Objective:**

To develop and validate a nomogram for predicting the risk of multidrug-resistant organisms (MDROs) infection in diabetic foot ulcer (DFU) patients.

**Methods:**

701 DFU patients were divided into training (491 cases) and validation (210 cases) sets (7:3 ratio). Multivariate logistic regression analysis was performed to identify the independent risk factors for MDRO infection in DFU patients. Two nomogram prediction models were developed based on the independent risk factors. The predictive efficacy of the prediction models was evaluated using the receiver operating characteristic (ROC) curve and calibration curve analysis. The decision curve analysis (DCA) was performed to evaluate the prediction model’s performance during clinical application.

**Results:**

Multivariate logistic regression analysis identified previous antibiotic therapy, surgical therapy, ulcer size>4cm^2^, and CRP as independent risk factors. Two models were developed and validated based on the analysis. Model 1 included previous antibiotic therapy, surgical therapy, and ulcer size>4cm^2^. Model 2 added a further laboratory indicator to Model 1, such as CRP. In the training set, the AUC of the nomogram for Model 1and Model 2 was 0.763(95% CI 0.711-0.815) and 0.789 (95% CI 0.740-0.838), respectively, and 0.837 (95% CI 0.744-0.900) and 0.845 (95% CI 0.785-0.905) in the validation set. The Youden indexes for Models 1and 2 were 0.416 and 0.470 in the training set and 0.558 and 0.588 in the validation set, respectively. Notably, Model 2 showed higher sensitivity and specificity. The calibration plot and Hosmer–Lemeshow test for Model 1 and Model 2 indicated that the predicted probability had good consistency with the actual probability in both the training set (P = 0.689 for Model 1 and P = 0.139 for Model 2) and validation set (P = 0.607 for Model 1and P = 0.635 for Model 2). The DCA curve indicated that the models had good clinical utility. All models performed well for both discrimination and calibration.

**Conclusion:**

This study developed two nomogram models for predicting MDRO infection risk in DFU patients. Model 2, with superior predictive performance, enables early identification of high-risk patients. These models facilitate targeted interventions, potentially reducing MDRO complications and healthcare burdens.

## 1 Introduction

Diabetes mellitus (DM) represents a major and escalating global health challenge. According to the International Diabetes Federation, the global prevalence of DM affected 463 million adults in 2019 and is projected to rise to 700 million by 2045 (1). Among the severe complications of diabetes, diabetic foot ulcer (DFU) has seen an alarming increase. Approximately 20% of individuals with diabetes will develop a DFU in their lifetime (2, 3), which is a leading cause of lower extremity amputation and significantly contributes to morbidity, mortality, and healthcare costs (4, 5), posing a particular threat in China (6).

A critical factor exacerbating the management and outcomes of DFU is infection. These ulcers are highly susceptible to bacterial colonization, often progressing to active infection (2, 7), with the rising incidence of multidrug-resistant organism (MDRO) infections compounding the problem. MDRO infections present a formidable clinical challenge, leading to delayed wound healing, elevated healthcare costs, and higher mortality compared to non-MDRO infections (8).

Although several studies have identified risk factors for MDRO infections in DFU patients (9–11), a significant translational gap persists. Existing evidence has not been synthesized into practical tools for real-time risk stratification, underscoring the urgent need for a robust methodology to predict MDRO infections early in this vulnerable cohort.

Risk prediction models offer a promising solution by integrating multiple predictors to quantify an individual’s probability of a specific outcome, thus aiding clinical decisions. While predictive models for endpoints like ulceration and amputation risk have advanced in diabetic foot care (12, 13). Few are specifically designed to forecast MDRO infection risk in DFU patients. This lack of targeted tools limits clinicians in identifying high-risk patients for early diagnosis or personalized treatment.

Therefore, this study aims to develop and validate a comprehensive risk prediction model for MDRO infection in DFU patients. By leveraging contemporary data and rigorous methods, we seek to create a clinically actionable clinically actionable tool to improve patient stratification, enable earlier intervention, and ultimately enhance clinical outcomes.

## 2 Research design and methods

### 2.1 Study population

**Figure 1:**
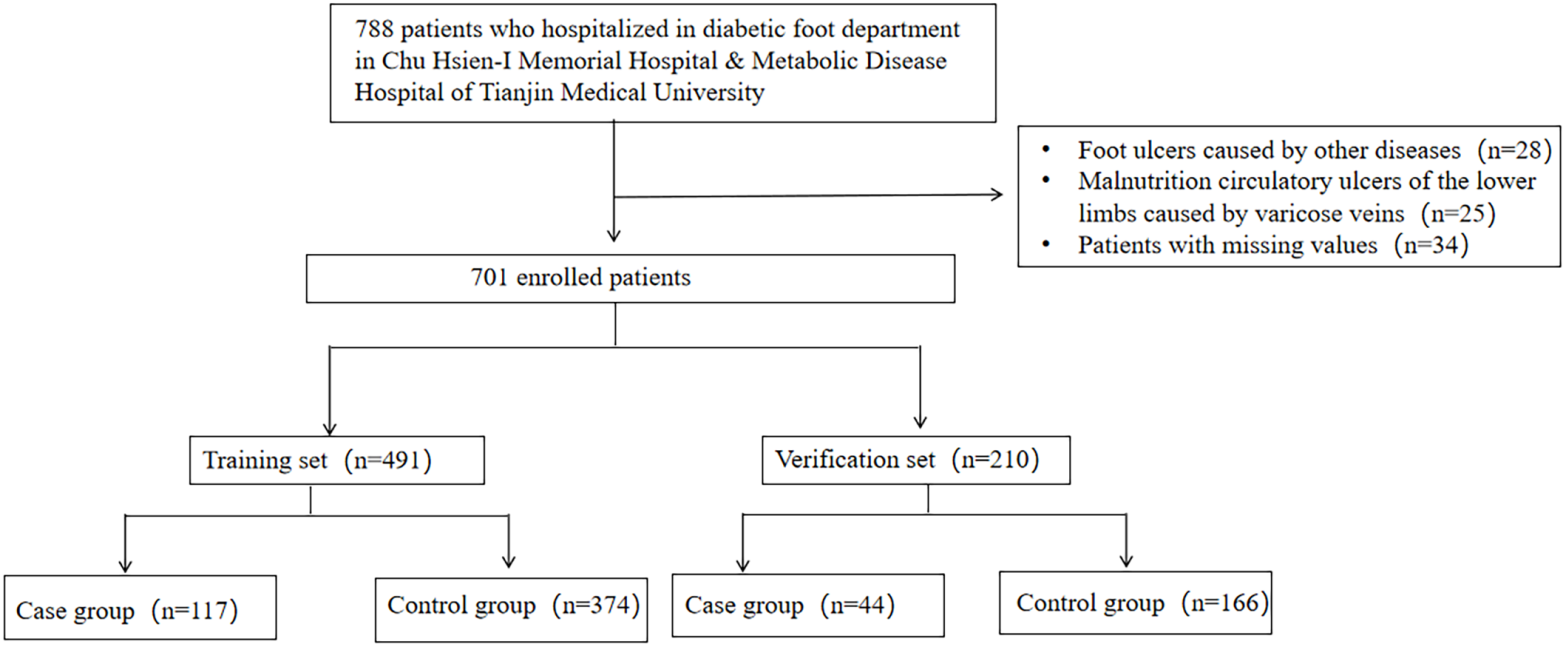
Study flow.

A total of 701 hospitalized patients with DFU in Chu Hsien-I Memorial Hospital & Metabolic Disease Hospital of Tianjin Medical University between May 2022 and November 2023were considered for this study. The research protocol was approved by the ethics committee of Tianjin Medical University Chu Hsien-I Memorial Hospital [ZXYJNYYKMEC2025-05]. Characteristics and the laboratory data were recorded at presentation including gender, age, diabetes duration, HbA1c and other biochemical data. On admission, specimens for culture were obtained after cleansing and debriding of the wound with a sterile cotton swab. Samples were promptly sent to the microbiology laboratory for culture in a sterile container. The strains were identified by VIETK mass spectrometry (bioMérieux, Marcy l’Etoile, France), and drug sensitivity test was conducted by VITEK 2-Compact system (bioMérieux, Marcy l’Etoile, France). Sensitivity tests were performed using the disc diffusion method to determine sensitivity according to the Clinical and Laboratory Standards Institute guidelines (CLSI M100, 32nd Edition) (14). MDROs were defined according to an international expert proposal set by the European Centre for Disease Prevention and Control (ECDC) and the Centers for Disease Control and Prevention (CDC) (15), characterized by acquired non-susceptibility to at least one agent in three or more antimicrobial categories, as determined by CLSI guidelines. The flow chart is shown in **Figure 1**.

### 2.2 Definition

Infection severity was defined according to the classification system of Infectious Diseases Society of America (IDSA) (16, 17). The Wagner system assessed ulcer depth and the presence of osteomyelitis or gangrene, using the following grades: grade 0 (pre‐ or post‐ulcerative lesion), grade 1 (partial/full‐thickness ulcer), grade 2 (probing to tendon or capsule), grade 3 (deep with osteitis), grade 4 (partial foot gangrene) and grade 5 (whole foot gangrene) (18). Previous antibiotic therapy was defined as the use of antibiotics within the preceding 30 days. Osteomyelitis was diagnosed based on a positive probing-to-bone test, abnormal plain X-ray, and abnormal laboratory tests (including erythrocyte sedimentation rate, high-sensitivity C-reactive protein, and procalcitonin) (19). Ischemia was defined by an ankle-brachial index <0.9, lower extremity CT angiography was performed when necessary. Peripheral arterial disease (PAD)was defined as the presence of stenosis or occlusion of lower limb arteries indicated by Doppler ultrasound (20). Diabetic kidney disease (DKD) was determined by a glomerular filtration rate (GFR) below 60 mL/min/1.73m^2^ or urinary albumin/creatinine ratio (ACR) above 30 mg/g for more than three months. Diabetic retinopathy (DR) was diagnosed by dilated fundus examination revealing microaneurysms or more serious lesions. Surgical therapy included both minor and major amputations.

### 2.3 Model development

The data of the 701 patients were divided into a training set (70% data, 491 cases) and a validation set (30% data, 210 cases) by random sampling in R software with “sample ()” function. All 491 patients’ data in the training set were analyzed for variable selection and risk prediction. Univariate logistic regression analysis was applied to select variables and those with p value < 0.1 were further screened in multivariate logistic regression. The selected independent clinical predictors(P<0.05) were used to establish the risk prediction model of MDRO infection in DFU patients, which was presented as Nomogram Model 1. Model 2 was built on the basis of Model 1 and included laboratory indicators.

### 2.4 Model validation

The performance of the prediction model was evaluated based on its discrimination ability, calibration ability, and clinical value. The receiver operating characteristic (ROC) curve was used to calculate the area under the curve (AUC) and its 95% confidence interval (CI). An AUC value closer to 1 indicates a higher prediction accuracy. Specifically, an AUC value above 0.7 suggests good discrimination ability. The calibration ability was assessed using a calibration plot accompanied by the Hosmer-Lemeshow test. The model was validated using the bootstrap method with 1000 resamples to quantify any overfitting. Additionally, decision curve analysis (DCA) was applied to evaluate the clinical utility of the nomogram based on its net benefits at different threshold probabilities.

### 2.5 Statistical analysis

Continuous variables were expressed as mean ± standard deviation (SD) or median values (interquartile range) and were assessed by independent group t-tests or Mann-Whitney U tests. Categorical variables were expressed as percentage (%) and were assessed by Chi-squared tests or Fisher’s exact test. Statistical analysis was carried out using SPSS 26.0 and R software (version 4.0.5, http://www.r-project.org). The R software was used to perform all the graphics based on R packages “foreign,” “rms,” “ggplot2,” “pROC,” and “glmnet”. P< 0.05 was considered statistically significant, except for the univariate logistic regression analysis, where P<0.1 was considered statistically significant.

## 3 Results

### 3.1 General data of the study subjects

A total of 788 patients were screened, with 87 excluded (28 non-diabetic ulcers, 25 varicose vein-related malnutritional circulatory ulcers, 34 missing data) (**Figure 1**). The remaining 701 DFU patients were randomly divided into a training set (70% of the data, 491 cases) and a validation set (30% of the data, 210 cases).Overall, 161 patients (23.0%) were diagnosed with MDRO infection. The training set included 117 (23.8%) MDRO infection patients and the validation set 44 (21.0%). No statistically significant differences were found in gender, age, diabetes duration, ulcer duration, etc. between training set and validation set (*P* > 0.05), indicating good comparability (**Table 1**).

**Table 1:** Baseline characteristics of patients in the training set and validation set.

Variable	Training set (n=491)	Validation set (n=210)	p‐value
MDRO	117(23.8%)	44(21%)	0.407
Age(years)	64.0 ± 11.7	63.2 ± 11.2	0.386
Gender (male/female)	374/117	166/44	0.407
DM duration(month)	188.4 ± 115.5	192.2 ± 107.9	0.682
Ulcer duration(weeks)	4(2-12)	4(2-12)	0.762
Previous antibiotic therapy (n,%)	287(58.5%)	124(59%)	0.883
Surgical therapy(n,%)	86(17.5%)	45(21.4%)	0.223
Ulcer type(n,%)			0.83
Neuropathic ulcer	149 (30.3%)	68 (32.4%)	
Ischemic ulcer	74 (15.1%)	29 (13.8%)	
Neuroischemic ulcer	268 (54.6%)	113 (53.8%)	
Ulcer size>4cm2(n,%)	254 (51.7%)	103 (49%)	0.515
Wanger grade			0.518
2 (n,%)	87 (17.7%)	35 (16.7%)	
3 (n,%)	357 (72.7%)	160 (76.2%)	
4 (n,%)	47 (9.6%)	15 (7.1%)	
Osteomyelitis (n,%)	305 (62.1%)	141 (67.1%)	0.205
Polymicrobial infection (n,%)	113 (23%)	47 (22.4%)	0.855
PAD (n,%)	346 (70.5%)	142 (67.6%)	0.452
Hypertension (n,%)	274 (55.8%)	112 (53.3%)	0.547
DR (n,%)	249 (50.7%)	124 (59%)	0.043
DKD (n,%)	244 (49.7%)	100 (47.6%)	0.615
HbA1c (%)	8.8 ± 2.1	8.9 ± 2.0	0.59
CRP (mg/L)	32.1(8.0-72.9)	29.3(5.9-74.9)	0.556
TC (mmol/L)	3.5 ± 1.8	3.3 ± 1.7	0.480
TG (mmol/L)	2.4 ± 1.7	2.5 ± 1.7	0.433
LDL-C(mmol/L)	3.4 ± 0.7	3.0 ± 0.9	0.448
HDL-C(mmol/L)	0.9 ± 0.3	0.9 ± 0.2	0.67
FIB(g/L)	5.5 ± 2.3	5.4 ± 2.0	0.787
D-Dimer(mg/L)	0.76(0.45-1.36)	0.72(0.48-1.17)	0.445

PAD, Peripheral artery disease; DR, Diabetic retinopathy; DKD,Diabetic kidney disease; HbA1c, glycated hemoglobin; CRP, C-reactive protein; TC, total cholesterol; TG, triglycerides; HDL-C, high-density lipoprotein cholesterol; LDL-C, low-density lipoprotein cholesterol; FIB, Fibrinogen.

### 3.2 Distribution of pathogens

A total of 715 strains of pathogenic bacteria were isolated, including 286 (40%) strains of gram-positive (GP) bacilli, 429(60%) gram-negative (GN) bacilli, and 44 (6.2%) other strains. *Staphylococcus aureus*, *Acinetobacter baumannii*, and *Pseudomonas aeruginosa* were the most frequently isolated from the foot ulcers. Predominant GP organisms included *Staphylococcus aureus*, *Staphylococcus epidermidis*, and *Enterococcus faecalis*, while the principal GN pathogens consisted of *Acinetobacter baumannii*, *Pseudomonas aeruginosa*, and *Klebsiella pneumoniae* (**Table 2**).

**Table 2:** Distribution of the pathogenic bacteria isolated from the DFUs.

Organisms	Number of strains (n)	Percentage (%)
Gram-positive bacteria	286	40
*Staphylococcus aureus*	132	18.5
*Staphylococcus epidermidis*	54	7.6
Other *Staphylococcus* spp.	22	3.1
*Enterococcus* spp.	43	6.0
*Streptococcus* spp.	35	4.9
Gram negative bacteria	429	60
*Escherichia coli*	37	5.2
*Enterobacter cloacae*	29	4.1
*Morganella morganii*	20	2.8
*Proteus* spp.	46	6.4
*Serratia marcescens*	14	2.0
*Acid producing Klebsiella*	23	3.2
*Klebsiella pneumoniae*	53	7.4
*Pseudomonas aeruginosa*	71	9.9
*Stenotrophomonas maltophilia*	9	1.3
*Acinetobacter baumannii*	83	11.6
Others	44	6.2

Among the isolated bacteria, 196 strains (27.0%) were identified as MDROs. This included 40 GP strains (accounting for 14.0% of all GP organisms) and 156 GN strains (comprising 36.4% of GN organisms).The top three strains with the highest MDRO rate were *Acinetobacter baumannii* (77%), *Escherichia coli* (54.1%), and *Enterobacter cloacae* (44.8%).It is also noteworthy that *Staphylococcus aureus* — the most frequently isolated pathogen — demonstrated an MDRO rate of 19.0%, while that of *Pseudomonas aeruginosa* was 26.8% (**Table 3**).

**Table 3:** MDROs distribution and drug resistance rate.

Bacteria	MDRO+	MDRO-	Resistant rate (%)
Gram-positive bacteria	40	246	14.0
*Staphylococcus aureus*	25	107	18.2
*Staphylococcus epidermidis*	5	49	9.3
Other *Staphylococcus* spp.	1	21	4.5
*Enterococcus* spp.	5	38	11.6
*Streptococcus* spp.	4	31	11.4
Gram negative bacteria	156	273	36.4
*Escherichia coli*	20	17	54.1
*Enterobacter cloacae*	13	16	44.8
*Morganella morganii*	4	16	20.0
*Proteus* spp.	6	40	13.0
*Serratia marcescens*	2	12	14.3
*Acid producing Klebsiella*	6	17	26.1
*Klebsiella pneumoniae*	15	38	28.3
*Pseudomonas aeruginosa*	19	52	26.8
*Acinetobacter baumannii*	64	19	77.1
Others	7	46	13.2
Total	196	519	27.4

### 3.3 Baseline data in the training set

The training set included 491 patients (117 in the MDRO+ group, 374 in the MDRO- group). There were no significant differences in age, sex, DM duration, or the level of HbA1c, TG, LDL-C, HDL-C and FIB. The percentage of polymicrobial infection, PAD, hypertension, and DR were also similar((P>0.05). Compared with the MDRO- group, the MDRO+ group showed higher levels of CRP (50.0 vs. 24.4 mg/L), D-Dimer (0.94 vs. 0.72 mg/L), and a lower level of TC (3.2 vs. 3.5 mmol/L). In the MDRO+ group, a higher prevalence of patients was observed with previous antibiotic therapy, surgical therapy, complications with DKD and larger ulcer size (**Table 4**).

**Table 4:** Baseline data in the training set.

Characteristics	MDRO+ (N = 117)	MDRO- (N = 374)	p‐value
Age(years)	62.1 ± 13.3	64.6 ± 11.1	0.069
Gender (male/female)	85/32	258/116	0.451
DM duration(month)	186.7 ± 108.0	188.9 ± 117.9	0.86
Ulcer duration(weeks)	4(3-20)	4(2-12)	0.014
Previous antibiotic therapy (n, %)	94(80.3%)	193(51.6%)	0.001
Surgical therapy (n, %)	54(46.2%)	32(8.6%)	0.001
Ulcer type (n, %)			0.009
Neuropathic ulcer	28 (23.9%)	121 (32.4%)	
Ischemic ulcer	11 (9.4%)	63 (16.8%)	
Neuroischemic ulcer	78 (66.7%)	190 (50.8%)	
Ulcer size>4cm2 (n, %)	84 (71.8%)	170 (45.5%)	0.001
Wanger grade			0.009
2 (n, %)	12 (10.3%)	75 (20.1%)	
3 (n, %)	98 (83.8%)	259 (69.3%)	
4 (n, %)	7 (6.0%)	40 (10.7%)	
Osteomyelitis (n, %)	88 (75.2%)	217 (58.0%)	0.001
Polymicrobial infection (n, %)	31 (26.5%)	82 (21.9%)	0.305
PAD (n, %)	89 (76.1%)	257 (68.7%)	0.128
Hypertension (n, %)	68 (58.1%)	206 (55.1%)	0.563
DR (n, %)	55 (47%)	194 (51.9%)	0.358
DKD (n, %)	68 (58.1%)	176 (47.1%)	0.037
HbA1c (%)	8.5 ± 1.9	8.9 ± 2.2	0.056
CRP (mg/L)	50.0(21.4-87.0)	24.4(4.3-71.1)	0.001
TC (mmol/L)	3.2 ± 1.6	3.5 ± 1.9	0.034
TG (mmol/L)	2.6 ± 1.9	2.3 ± 1.6	0.191
LDL-C(mmol/L)	4.2 ± 1.9	3.1 ± 1.0	0.386
HDL-C(mmol/L)	0.9 ± 0.2	0.9 ± 0.3	0.188
FIB(g/L)	5.5 ± 2.0	5.5 ± 2.4	0.922
D-Dimer(mg/L)	0.94(0.56-1.74)	0.72(0.44-1.30)	0.005

### 3.4 Predictor selection and model development

We calculated the risk factors by univariable and multivariable logistic regression (**Table 5**). The univariable regression analysis showed that age, ulcer duration, CRP, D-Dimer, previous antibiotic therapy, surgical therapy, ulcer type, ulcer size >4cm^2^, Wanger grade, osteomyelitis, and DKD were significant influencing factors for the occurrence of MDRO infection in DFU patients (P < 0.05). Based on the univariable analysis with a significance level of P < 0.1, we included the following 11 variables: age, ulcer duration, previous antibiotic therapy, surgical therapy, ulcer type, ulcer size, Wanger grade, osteomyelitis, DKD, CRP, and D-Dimer. The analysis results demonstrated that previous antibiotic therapy, surgical therapy, ulcer size>4cm^2^, and CRP are independent influencing factors for the occurrence of MDRO infection in DFU patients. The variables identified by multivariate regression analysis were used to establish the risk model. Based on the multivariate regression analysis, we established two prediction models. Model 1(excluding laboratory indicators):previous antibiotic therapy, surgical therapy, and ulcer size>4cm^2^. Model 2 (including laboratory indicators):previous antibiotic therapy, surgical therapy, ulcer size>4cm^2^, and CRP.

**Table 5:** Univariate and multivariate logistic regression analyses in the training set.

Variable	Univariable analysis		Multivariable analysis	
	HR (95%)	p‐value	HR (95%)	p‐value
Age(years)	0.982(0.965-1.000)	0.046		
Gender	1.194(0.753-1.895)	0.451		
DM duration(month)	1.000(0.998-1.002	0.86		
Ulcer duration(weeks)	1.013(1.000-1.025)	0.049		
Previous antibiotic therapy (n, %)	3.833(2.327-6.313)	0.001	2.316(1.343-3.994)	0.003
Surgical therapy (n, %)	9.161(5.483-15.304)	0.001	7.359(4.249-12.745)	0.001
Ulcer type (n, %)				
Neuropathic ulcer	0.564(0.346-0.919	0.021		
Ischemic ulcer	0.425(0.213-0.850)	0.016		
Neuroischemic ulcer	REF			
Ulcer size>4cm2 (n, %)	3.055(1.945-4.796)	0.001	1.790(1.065-3.008)	0.028
Wanger grade				
2 (n, %)	REF			
3 (n, %)	2.365(1.232-4.540)	0.01		
4 (n, %)	1.094(0.399-2.997)	0.862		
Osteomyelitis (n, %)	2.195(1.376-3.503)	0.001		
Polymicrobial infection (n, %)	1.284(0.796-2.070)	0.306		
PAD (n, %)	1.447(0.898-2.333)	0.129		
Hypertension (n, %)	1.132(0.744-1.723)	0.564		
DR (n, %)	0.823(0.543-1.248)	0.359		
DKD (n, %)	1.561(1.026-2.375)	0.037		
HbA1c (%)	0.910(0.821-1.009)	0.072		
CRP (mg/L)	1.004(1.001-1.007)	0.007	1.004(1.001-1.008)	0.011
TC (mmol/L)	0.890(0.792-1.000)	0.05		
TG (mmol/L)	1.031(0.912-1.166)	0.148		
LDL-C(mmol/L)	1.021(0.981-1.063)	0.299		
HDL-C(mmol/L)	0.561(0.238-1.326)	0.188		
FIB(g/L)	1.005(0.918-1.099)	0.922		
D-Dimer(mg/L)	1.189(1.023-1.382)	0.024		

### 3.5 Model discrimination

**Figure 2:**
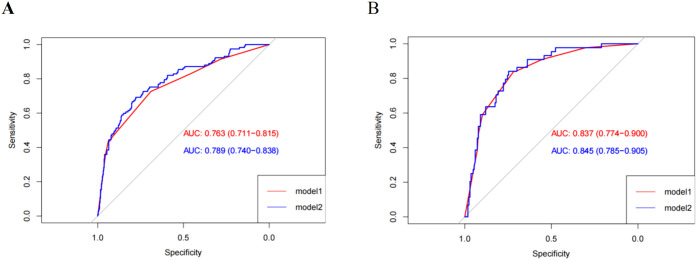
Comparison of the ROC curves of the nomograms for MDRO infection possibility prediction in the training set **(A)**, and in the validation set **(B)**.

**Table 6** shows the area under the ROC curve and the performance of each model for the training and validation sets. The ROC curves are shown in **Figure 2**. By internal bootstrap validation with 1000 resamples, the mean AUC of the nomogram based on the training set was 0.763 (95% CI 0.711-0.815) for Model 1 and 0.789(0.740-0.838) for Model 2, respectively (**Figure 2A**), indicating good discrimination ability for predicting the risk of MDRO infection. The accuracy of the nomogram in the validation set was similar to that of the training group, with AUC values of 0.837(95% CI 0.744-0.900) for Model 1 and 0.845 (95% CI 0.785-0.905) for Model 2, respectively (**Figure 2B**). The Youden indexes for Models 1 and 2 were 0.416 and 0.470 in the training set and 0.558 and 0.588 in the validation set, respectively. Overall, the two nomograms showed good predictive accuracy in estimating the risk of MDRO infections in both the training and validation sets.

**Table 6:** Prediction performance of the nomograms for estimating the risk of MDRO in DFU patients.

Prediction Performance Indicator	Model 1		Model 2	
	Training set	Validation set	Training set	Validation set
AUC (95% CI)	0.763(0.711-0.815)	0.837(0.744-0.900)	0.789(0.740-0.838)	0.845(0.785-0.905)
Youden index	0.416	0.558	0.47	0.588
Sensitivity, %	72.6	84.1	69.2	84.1
Specificity, %	69	71.7	77.8	74.7

### 3.6 Model calibration

**Figure 3:**
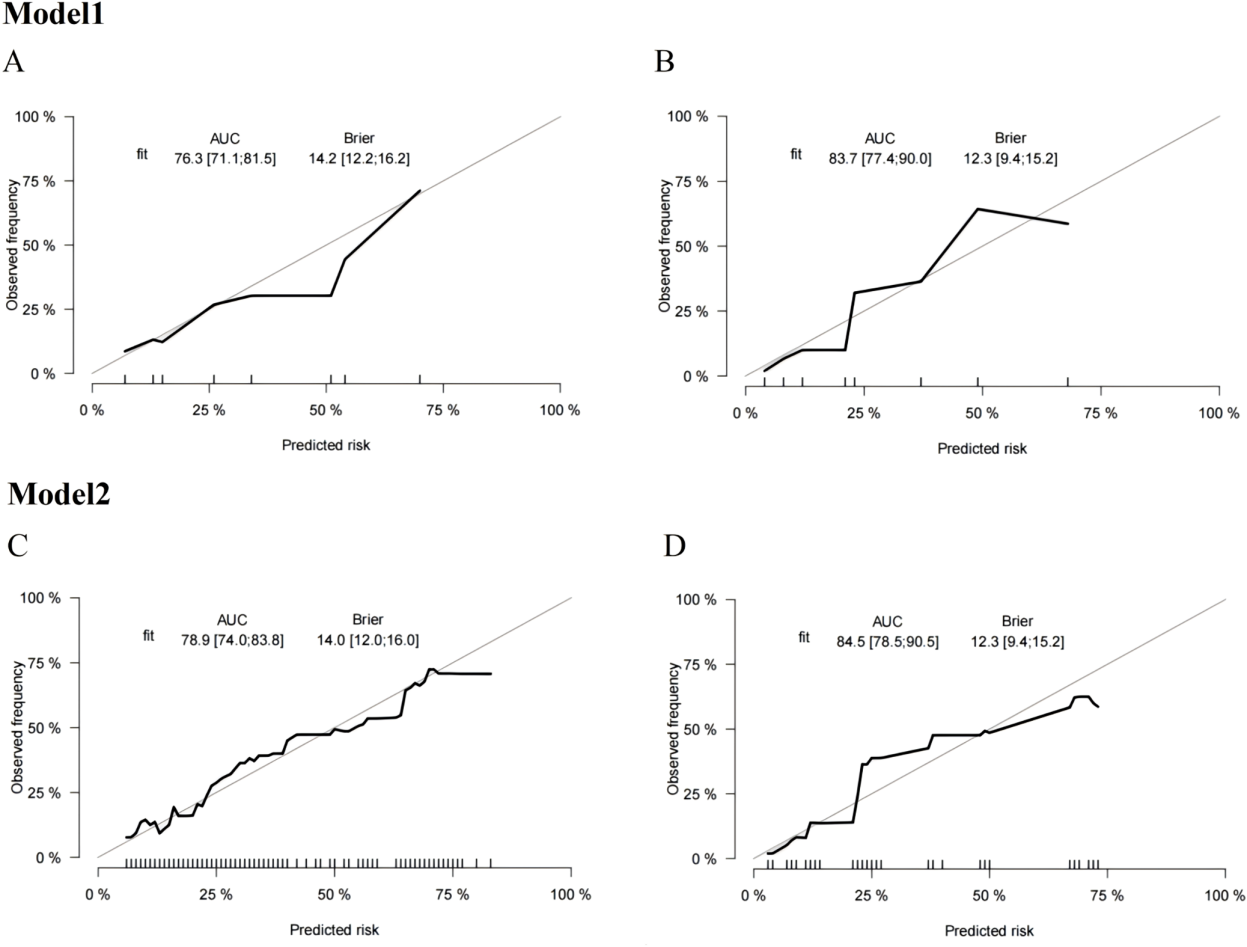
Model1:Calibration plots of the nomograms for MDRO infection prediction of the training set **(A)** and validation set **(B)**. Model2:Calibration plots of the nomograms for MDRO infection prediction of the training set **(C)** and validation set **(D)**.

In the internal bootstrap validation of the training set, both Model 1 and Model 2 showed good consistency between the predicted probability and the actual probability, as demonstrated by the calibration plots (**Figures 3A, C**) and the Hosmer-Lemeshow tests (P = 0.689 for Model 1 and P = 0.139 for Model 2). Similarly, in the validation set, the calibration plots (**Figures 3B, D**) and the Hosmer-Lemeshow tests (P = 0.607 for Model 1 and P = 0.635 for Model 2) indicated that both Prediction Model 1 and Prediction Model 2 fit well.

### 3.7 Evaluation of clinical applicability of nomograms

**Figure 4:**
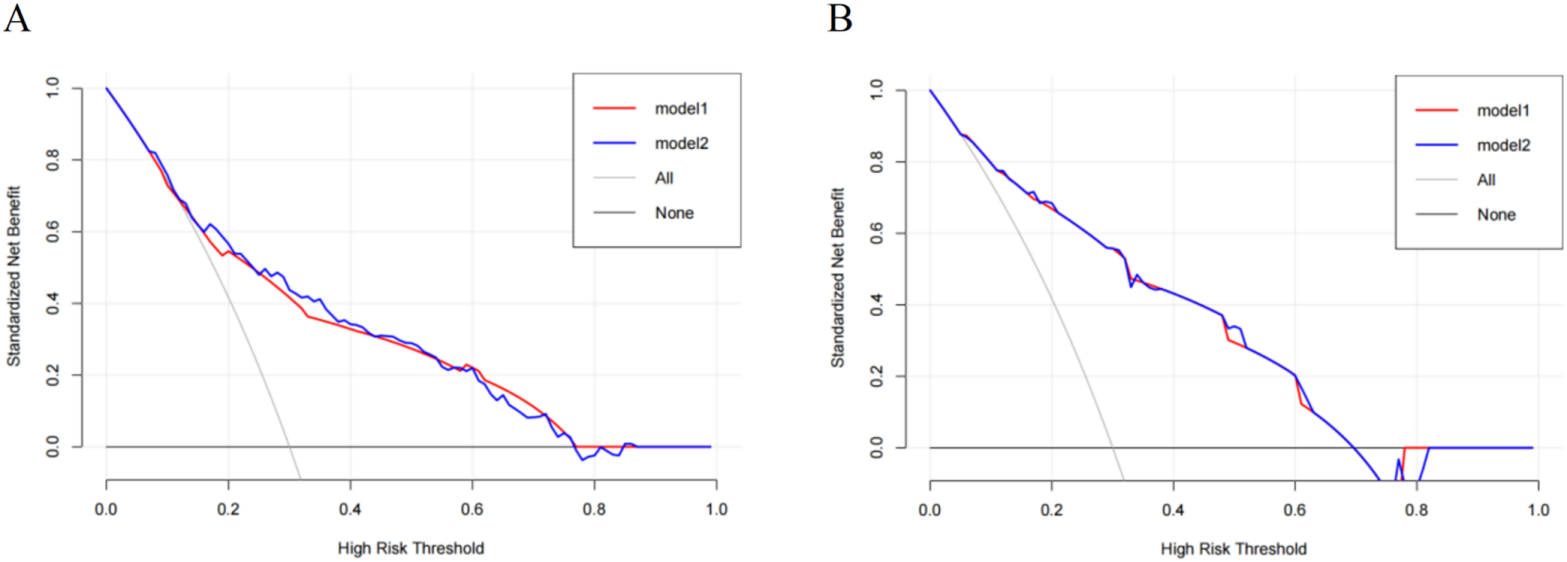
Decision curve analysis for Models 1 and 2: **(A)** Training set, **(B)** Validation set.

We applied decision curve analysis (DCA) to evaluate the clinical utility of the models based on their net benefits at different threshold probabilities. The y-axis measured the net benefit. The black solid line represented the assumption that all patients were without MDRO infection and received a treat-none strategy. The gray solid line represented the assumption that all patients had MDRO infection and received a treat-all strategy. The red and blue solid line represented the net benefit of our prediction model 1 and model 2, respectively. The DCA curve in the training set (**Figure 4A**) showed that if the threshold probability of patients was between 18% and 78%, our prediction models resulted in a superior net benefit compared to the treat-none or treat-all strategies. In the validation set, the prediction models were useful when the absolute risk thresholds were between 10% and 70%, with Model 2 demonstrating a higher net benefit (**Figure 4B**).

### 3.8 Nomograms

**Figure 5:**
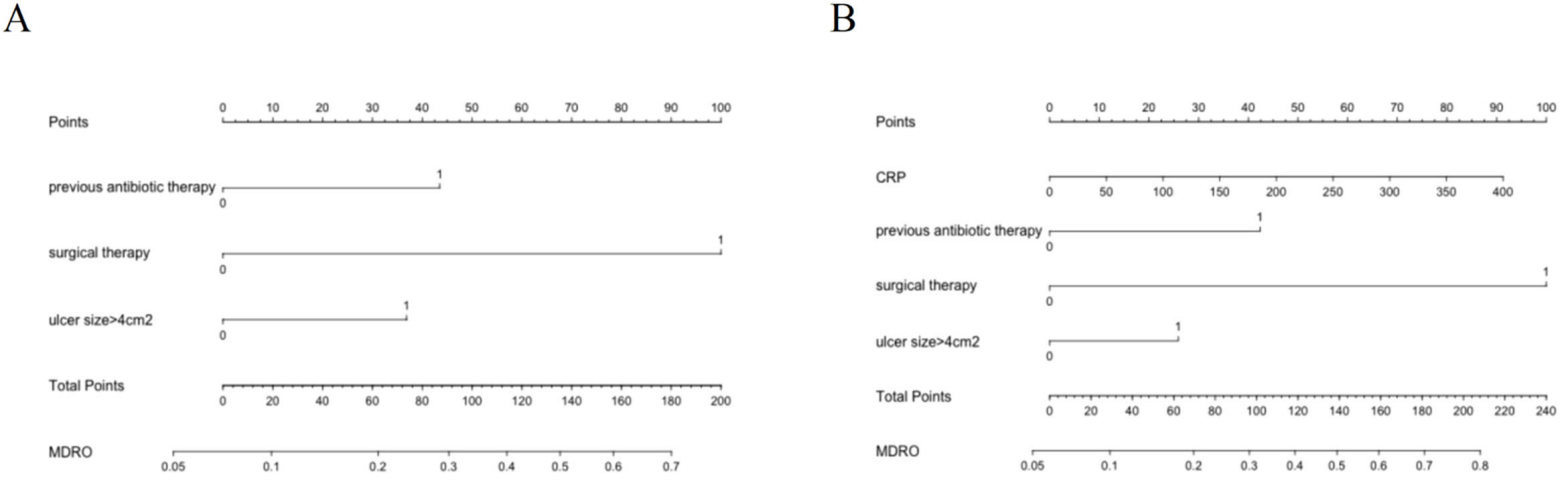
Nomogram for **(A)** Model 1, **(B)** Model 2.

The nomogram provides a convenient tool to help doctors judge the risk of MDRO infection (**Figure 5**). The nomogram of Model 1 includes previous antibiotic therapy, surgical therapy and ulcer size>4cm^2^ (**Figure 5A**). The nomogram for Model 2 includes all variables in Model 1 and laboratory indicator CRP (**Figure 5B**). To use the nomogram, mark the value of each included factor on the corresponding axis. Draw a vertical line from each value to the top line to obtain corresponding points. Sum the points from each variable value. Locate the total points on the scale and project it vertically onto the bottom axis to obtain the risk of MDRO infection.

## 4 Discussion

Diabetic foot infections (DFIs) are a common clinical challenge, and antibiotic treatment is typically guided by wound secretion cultures. However, bacterial cultures often require 18 to 24 hours, and certain pathogens, such as fungi, may take even longer to identify. For routine bacterial cultures, laboratory reports are generally issued within 3 to 5 days, making it difficult to identify MDROs early and manage them promptly. Therefore, early and timely identification and management of MDROs are critical. This study, based on data from DFU patients in Tianjin, China, successfully developed and validated two non-invasive nomogram models (Model 1 and Model 2), providing a dynamic risk assessment tool for clinical use.

In our study, a total of 715 pathogenic strains were isolated. Of the cultured bacteria, *Staphylococcus aureus* was the most frequently identified organism, which aligns with findings from broader studies on pathogen distribution in China (21). Among these isolates, MDROs accounted for 27%, a proportion lower than that reported in a related study conducted at our hospital in 2022. Nevertheless, *Acinetobacter baumannii* continued to demonstrate the highest resistance rate, highlighting the ongoing severity of challenges in MDRO control (9).

This study identified the previous antibiotic therapy, surgical therapy, ulcer size>4cm^2^ and CRP level as independent risk factors for MDRO infections. The long-term and repeated use of antimicrobial drugs can induce mutations in pathogen resistance genes and the emergence of multidrug resistance gene complexes (8, 22, 23). Surgical intervention may increase MDRO infection risk by disrupting local tissue barriers, inducing immunosuppression, and promoting biofilm formation (9). Diabetic patients often experience a decline in immune function, which facilitates the spread of opportunistic pathogens. Saltoglu et al. (24) demonstrated that invasive procedures significantly impair host defense mechanisms, creating opportunities for resistant pathogen colonization. Additionally, patients with larger ulcer area are more prone to polymicrobial infections, further exacerbating MDRO infection risk (10). CRP is a marker of infection and inflammation, which increases in level during bacterial infections (25). Elevated CRP levels reflect systemic inflammation and bacterial load, which may enhance MDRO colonization by disrupting local immune. It not only reflects infection severity but is also associated with MDRO resistance gene regulation. Wang et al. (26) found that CRP >50mg/L is an independent predictor of MDRO infections in ICU patients, and our study further validated its generalizability in the DFU population.

Ma et al. (27) developed a static scoring model for MDRO risk prediction, it did not differentiate clinical stages. Based on the results of univariate and multivariate logistic regression analyses within the training set, we successfully developed a staged nomogram (Model 1 and Model 2) with greater clinical flexibility. Model 1 enables initial risk assessment using simple clinical parameters (e.g., previous antibiotic use, surgical history, and ulcer size), whereas Model 2 enhances predictive precision with the integration of CRP (AUC increased from 0.763 to 0.789). So in clinical practice, Model 1 is suitable for initial screening in outpatient settings, while Model 2 can optimize decision-making after laboratory results are available, providing a flexible risk management strategy.

Several limitations should be noted in this study. First, the single-center, retrospective design may introduce inherent selection bias and restricts the external validity of our nomograms. Future validation in multi-center, geographically diverse cohorts is essential. Second, the models were internally validated but lacked external validation in an independent cohort; therefore, their general applicability requires further confirmation. Third, the absence of detailed antibiotic susceptibility data in this study may limit the model’s clinical applicability, making it difficult to inform precise therapeutic decisions therapeutic decisions. Future research integrating complete microbiological data will be crucial for determining whether drug resistance plays a key mediating role in the pathway from risk factors to adverse outcomes. Fourth, the model was constructed using a limited set of predictors, and we did not incorporate certain significant variables such as nutritional status indicators (e.g., albumin). This omission could affect the generalizability and accuracy of the nomogram. Prospective studies should incorporate standardized nutritional assessments to validate our findings and provide more precise therapeutic guidance. Lastly, a cost-effectiveness analysis was not conducted in this study. Consequently, its true effect on clinical endpoints and medical expenditures has yet to be determined and should be verified through prospective implementation research.

Despite these limitations, this study successfully developed and internally validated the nomograms specifically designed for predicting MDRO infection risk in patients with DFU. It provides a solid methodological foundation for future efforts aimed at external validation, clinical implementation, and technological integration.

## 5 Conclusions

To summarize, this study developed two nomogram models for predicting MDRO infection risk in DFU patients, which have high clinical practicality. Model 2 offers a more specific prediction method due to its best performance in predicting the risk of MDRO infection among the two models. By enabling early identification of high-risk patients and facilitating targeted interventions (e.g., enhanced isolation or optimized antibiotic strategies), these models have the potential to reduce MDRO infection-related complications and healthcare burdens.

## Data Availability

The raw data supporting the conclusions of this article will be made available by the authors, without undue reservation.
